# Protocol for an OpenSAFELY cohort study collecting patient-reported outcome measures using the TPP Airmid smartphone application and linked big data to quantify the health and economic costs of long COVID (OpenPROMPT)

**DOI:** 10.1136/bmjopen-2022-071261

**Published:** 2023-02-17

**Authors:** Emily Herrett, Keith Tomlin, Liang-Yu Lin, Laurie A Tomlinson, Mark Jit, Andrew Briggs, Michael Marks, Frank Sandmann, John Parry, Christopher Bates, Jessica Morley, Seb Bacon, Benjamin Butler-Cole, Viyaasan Mahalingasivam, Alan Dennison, Deb Smith, Ethan Gabriel, Amir Mehrkar, Ben Goldacre, Liam Smeeth, Rosalind M M Eggo

**Affiliations:** 1 London School of Hygiene and Tropical Medicine, London, UK; 2 Hospital for Tropical Diseases, London, UK; 3 TPP, Leeds, UK; 4 Bennett Institute for Applied Data Science, University of Oxford, Oxford, UK; 5 Patient and Public Involvement Steering Committee, London, UK; 6 Institute of Applied Health Research, University of Birmingham, Birmingham, UK

**Keywords:** COVID-19, HEALTH ECONOMICS, Quality of Life

## Abstract

**Introduction:**

The impact of long COVID on health-related quality of-life (HRQoL) and productivity is not currently known. It is important to understand who is worst affected by long COVID and the cost to the National Health Service (NHS) and society, so that strategies like booster vaccines can be prioritised to the right people. OpenPROMPT aims to understand the impact of long COVID on HRQoL in adults attending English primary care.

**Methods and analysis:**

We will ask people to participate in this cohort study through a smartphone app (Airmid), and completing a series of questionnaires held within the app. Questionnaires will ask about HRQoL, productivity and symptoms of long COVID. Participants will be asked to fill in the questionnaires once a month, for 90 days. Questionnaire responses will be linked, where possible, to participants’ existing health records from primary care, secondary care, and COVID testing and vaccination data. Analysis will take place using the OpenSAFELY data platform and will estimate the impact of long COVID on HRQoL, productivity and cost to the NHS.

**Ethics and dissemination:**

The Proportionate Review Sub-Committee of the South Central—Berkshire B Research Ethics Committee has reviewed and approved the study and have agreed that we can ask people to take part (22/SC/0198). Our results will provide information to support long-term care, and make recommendations for prevention of long COVID in the future.

**Trial registration number:**

NCT05552612.

**STRENGTHS AND LIMITATIONS OF THIS STUDY**

- OpenPROMPT will recruit people through a free app that is already in use within the National Health Service (NHS), meaning that the results will be stored in the safest possible way through NHS accredited data centres.
- Analysis will be through OpenSAFELY, which—as a Trusted Research Environment—will ensure participant confidentiality.
- Participants are able to self assert their identity through the NHS app, which links to the Airmid app, ensuring patient identity without imposing on care providers.
- The advantage of OpenPROMPT is that it collects information directly from participants and links to the rich routine data held in the electronic health record.
- An important limitation is that participants in the study will be self-selected, which means their experience of long COVID may not be representative of all people with long COVID.

## Introduction

### Long COVID in the UK

There is a growing evidence about the sequelae of COVID-19 infection.^1^ Long COVID-19, or ‘post-COVID syndrome’ includes any symptoms after 12 weeks of infection not explained by another diagnosis.^2^ It is anticipated that long COVID-19 will represent a large part of the ultimate impact of the COVID-19 pandemic, both in terms of its long-lasting health effects^3^ and economic costs generated through impacts on health-related quality of life (HRQoL), productivity,^4^ especially on working-age adults who are ill or caring for those who are ill, and increased use of healthcare resources.^5^ However, the size of this burden has not been comprehensively quantified.

In November 2022, persistent symptoms at least 12 weeks after infection are estimated to affect at least 2.1 million people in the UK (3.3% of the population).^6^

### Mitigating the impact of long COVID

To mitigate the impact of long COVID, it is vital to collect information that will enable the National Health Service (NHS) to make healthcare decisions, evaluate and prioritise vaccination boosters and other COVID-19-related interventions. First, interventions used by the NHS are evaluated for cost-effectiveness by the National Institute for Health and Care Excellence. This requires quantitative information about impacts measured using (1) standardised measures of changes in patient HRQoL (including physical and mental health effects) using the EuroQoL EQ-5D instrument and (2) formal quantification of the healthcare burden of the condition and its treatment. In addition, evaluations by other organisations often also require (3) measures of society-level impacts such as decreases in productivity in people with the condition. Second, patient-reported outcome measures such as HRQoL are vital for monitoring the effectiveness of interventions funded by the NHS, as well as to guide clinical decision making.

### Gaps in the evidence

There is a growing evidence about the health economic impact of COVID and its sequelae, with lower quality of life scores reported among those with COVID, post-COVID syndrome or long COVID, compared with those without COVID or without ongoing symptoms.^7–9^

However, a comprehensive analysis of HRQoL in long COVID should take account of changes in the circulating strain of COVID-19, the impact of vaccination, time after COVID-19 and should compare those with long COVID to those not experiencing ongoing symptoms. To date, the existing literature has not explored HRQoL using the validated EQ-5D instrument among those living with long COVID in England in such depth. This means that the impact of long COVID cannot be reliably included in future planning for COVID epidemics, nor can we accurately measure the true scale of its effects or burden on the NHS, or the cost-effectiveness of interventions to mitigate these effects. Determining how long COVID affects HRQoL and NHS costs is critical for evaluation and for planning the future of COVID response in the UK. This is particularly true in light of some evidence suggesting a reduced risk in vaccinated individuals since the emergence of the Omicron strain of SARS-CoV-2,^10^ while the risk of long COVID increases with the number of (re)infections.^11^ Lastly, with a focus on long-term disability in people living with long COVID, the study is also responding directly to the research needs identified by WHO in October 2022.^7^

To address these gaps in the evidence, this study will use novel methods to assess the impact of long COVID on HRQoL, healthcare utilisation, cost to the NHS and productivity among both hospitalised and non-hospitalised COVID cases, taking account of the timing of COVID diagnosis, and the number and timing of infections and vaccinations. We will also investigate the effect of demographic and clinical factors, and perform a health economic evaluation of future vaccination strategies, incorporating the impact on long COVID.

## Methods and analysis

### Study design and setting

OpenPROMPT is a cohort study among adults in England, recruiting between November 2022 and May 2023. This study is being conducted in collaboration with TPP, a general practitioner (GP) software provider. TPP’s software system is called SystmOne and holds patient electronic health records for 40% of the population of England. Their in-house smartphone app, Airmid,^12^ has been developed as a patient-facing mobile application, and links directly to SystmOne records. It was created to allow appointment booking, for patients to access their own medical records, and for patients to input their own data to feed into their medical record. Airmid’s Research Module can run surveys and bespoke questionnaires, with results recorded in a patient’s Personal Health Record. Airmid requires either a NHS Login or TPP SystmOnline Username and Password. Any individual can download the Airmid app, even if their GP does not use TPP software. Airmid and SystmOne are being provided by TPP for no charge.

### Inclusion criteria

Any adult in England can take part in the study (irrespective of long COVID/COVID-19 status), if they are able to read and understand English, and download and use the Airmid app (available on iOS and Android free of charge).

### Recruitment

For people who already have the app installed on their device, the study page will be visible when they log in to the app, and they will be able to enrol in the study. The study will also be advertised to people registered at general practices that use SystmOne software. GPs at these practices will send text messages or emails to all adults in the system (who they have permission to contact). The text message or email will contain a link to the study website. This will hold information on the study and a guide to participation, through the Airmid app. The guide will include a link to instructions on how to download the Airmid app and where to find the study within the app. The study will be advertised more broadly through social media platforms to long COVID groups and the general population. Potential participants will be invited to download the app and opt-in to the study.

Potential participants will be asked to read the patient information sheet and consent statements within the app, and will move an ‘opt-in’ slider within the app to provide an affirmative response that they have read and understood the information, and agree to the consent statements. Consent will be recorded and dated within the participant’s personal health record.

We will monitor recruitment weekly and compare against our targets for recruitment. We will repeat efforts to increase recruitment through social media campaigns in the first week of each month during the study period.

### Study questionnaires

The study questionnaires will be available within the Airmid app once a participant has opted-in to the study. There are two study-specific questionnaires, enquiring about participants’ demographic characteristics, and about their experience of COVID-19, long COVID and COVID-19 vaccination. The study also asks participants to complete four validated questionnaires: (1) EuroQoL EQ-5D 5L,^13^ measuring HRQoL and enabling calculation of quality-adjusted life-years lost due to acute COVID and long COVID; (2) the Work Productivity and Activity Impairment Questionnaire: Specific Health Problem 2.0, measuring productivity and estimating the economic value of productivity loss in people with long COVID^14^; (3) the MRC Dyspnoea scale measuring breathlessness^15^ and (4) the FACIT (Functional Assessment of Chronic Illness Therapy) tool, measuring fatigue.^16^ These symptom questionnaires will allow valid symptom comparisons between those with and without long COVID.

### Data linkage

For participants who are registered at a general practice that uses TPP SystmOne software (around 40% of English general practices),^17^ OpenPROMPT will link participant questionnaire data to the OpenSAFELY-TPP platform: initially to enhance the existing OpenSAFELY-TPP data with results of questionnaires, then to analyse individual-level data and translate the impacts to the population level. OpenSAFELY-TPP is a secure platform for analysis of electronic patient health records and is a Trusted Research Environment with strict governance to maximise patient confidentiality and privacy. The main purpose of OpenSAFELY-TPP is to conduct research in the public interest to deepen our understanding of the COVID-19 pandemic. OpenSAFELY-TPP will incorporate the questionnaire data into the existing OpenSAFELY framework. The data will be linked to existing data and infrastructure via strongly pseudonymised NHS number, based on industry standard encryption. Access to data within the OpenSAFELY TPP platform is under the COVID-19 Control of Patient Information (COPI) Regulation 3. This means that data can be processed within OpenSAFELY, without the need for each GP practice to consent. Additionally, OpenPROMPT will seek informed patient-level consent for analysis of study-specific data, with the aim that after the withdrawal of COPI the study data will remain accessible.

OpenSAFELY-TPP holds linked data including primary care, COVID-19 testing, hospitalisations, COVID-19 Blueteq treatment data and mortality data. Primary care data from OpenSAFELY will link to coded consultations with the general practice team, diagnosis codes, prescriptions, referral codes and tests undertaken in primary care.

**Figure 1:**
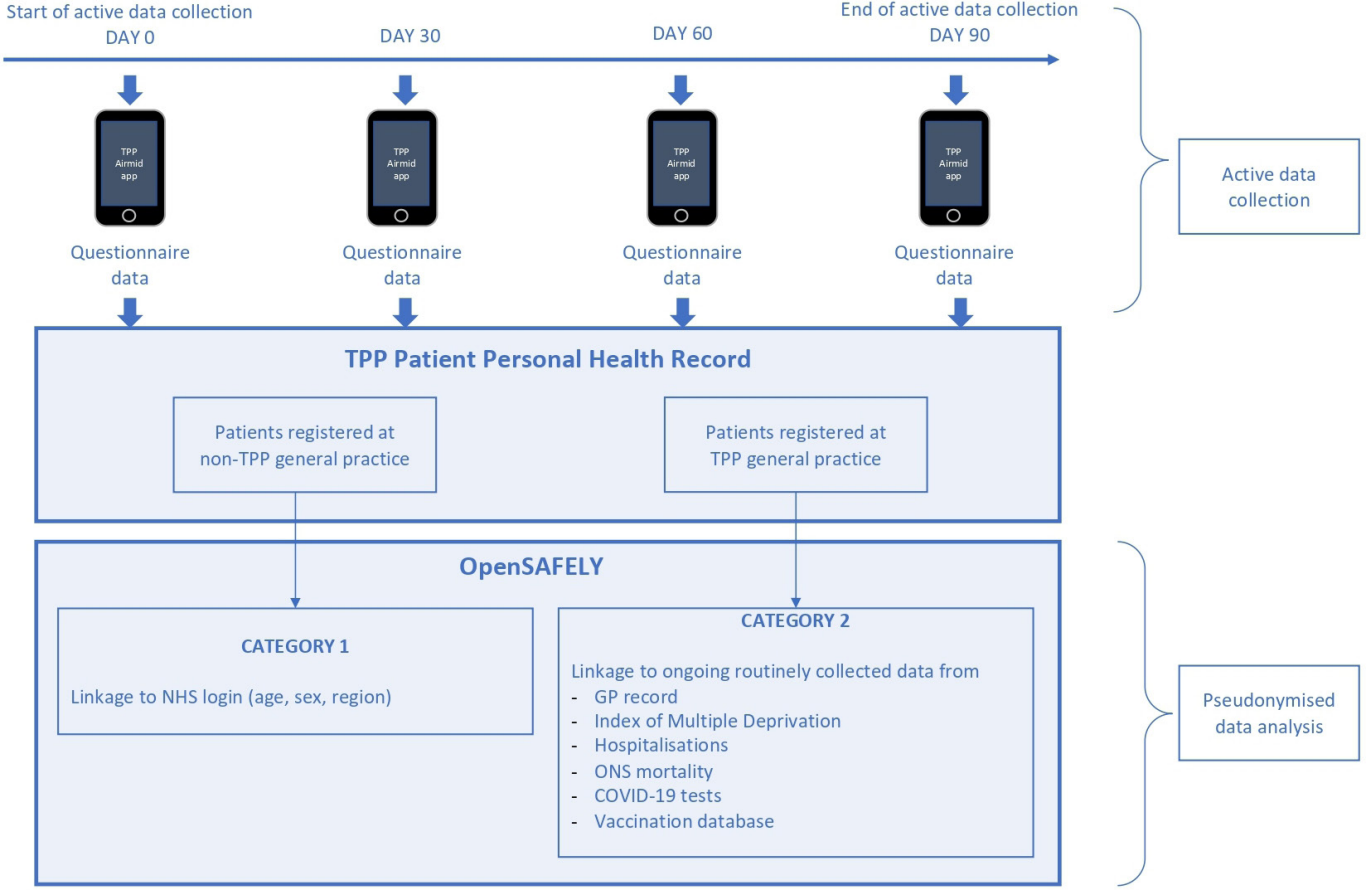
Overview of study. GP, general practitioner; NHS, National Health Service; ONS, Office for National Statistics; TPP refers to TPP SystmOne clinical software.

For participants who are not registered at a practice using TPP SystmOne software, the study data will be limited to the questionnaire results, and analysis will take place outside the OpenSAFELY environment. Figure 1 provides an overview of the study.

### Follow-up

Participants will be prompted by a push notification (a pop up on their phone screen) to respond to each questionnaire at the time of recruitment, and then at 30, 60 and 90 days after recruitment. The demographic questionnaire will be asked only at baseline (including eg, age, sex, relationship status, socioeconomic status). Participants will be invited to complete all questionnaires, but partially complete data will also be available for analysis.

System reports will flag incomplete questionnaires and one additional push notification reminder will be sent to participants who have not completed any questionnaires within 5 days of the initial notification. Active follow-up will end at 90 days when participants are asked to complete their final set of questionnaires. Ongoing, passive follow-up will be continued within the routinely collected electronic health record once the data linkage is in place. At the point of recruitment, participants will be offered the opportunity to consent to linkage and use of their data by researchers.

Participants are free to withdraw from the study at any time and there will be no implications for their care. Withdrawal of consent will occur through Airmid, by moving the opt-in toggle to opt-out.

### Study size

The primary research question is to estimate the impact of long COVID on HRQoL as measured using EQ-5D scores at multiple timepoints. Average quality of life in a study of people with severe hospitalised COVID estimated pre-COVID HRQoL scores (EQ-5D) to be 0.9655 (SD 0.0958)) and then 0.7054 (SD 0.2514) 6 months after COVID.^18^ We anticipate that people with long COVID participating in the present study will be a mixture of hospitalised and non-hospitalised patients, so are likely to be affected to a lesser extent than a cohort of only hospitalised patients. A second study in a general population cohort estimated EQ-5D scores to be 0.979 (SD 0.0530) among people with no chronic diseases, and 0.9360 (SD 0.112) among those with one chronic disease.^19^

We have therefore performed a power calculation based on the conservative estimates of 0.979 in participants without long COVID, and 0.9360 in those with long COVID (comparable to people with one chronic disease). At the time of calculation, population prevalence of long COVID was estimated to be 2.7%. We assumed that we will over-recruit people with long COVID, due to our focused recruitment strategy. Assuming that 5% of our participants have long COVID, this gives a sample size of 1172 participants to achieve 80% power (alpha 0.05) (corresponding to 1116 in the group without long COVID and 56 among those with long COVID). We intend to keep recruiting beyond this target, as there is no additional cost to the study and minimal risk to the additional respondents, and it will increase our power to address future research questions.

To explore sampling bias, we will compare characteristics of people registered at GPs using TPP SysmOne software who take part in the study to those who do not take part, using data from their electronic health record, and additionally compare our results to those of the Office for National Statistics^20^ and the Institute for Fiscal Studies.^21^

### Analysis plan

The main outcome will be QALYs lost after an episode of COVID-19, calculated from HRQoL as measured by the EuroQOL EQ-5D questionnaire at multiple timepoints. OpenPROMPT will also explore outcomes based on the results from the symptom and productivity questionnaire results.

The main exposure will be long COVID. This will be self-reported by participants in the questionnaires, and recorded in routinely collected data in OpenSAFELY (primary and secondary care data and COVID-19 testing data). The definition of long COVID used in this study will be based on a combination of self-report, diagnoses and referrals for long COVID; the definition will be explored with sensitivity analyses.

OpenPROMPT will analyse individual-level determinants of HRQoL deficits in people with long COVID-19, including key variables such as ethnicity, age, socioeconomic status and pre-existing health conditions.

This will generate a strong health economic evidence base for assessing the impact of long COVID and evaluating future interventions (including essential inputs to cost-effectiveness analyses of interventions like vaccination boosters, therapeutics and non-pharmaceutical interventions), and demonstrate the effect of long COVID on the health system so far.

### Patient and public involvement

We have two streams of patients and public involvement. First, OpenPROMPT recruited a three person steering group, including members with long COVID, those experienced in patient and public involvement or with an interest in long COVID. The steering group aims to meet with the research team online every 6 months. This group has given input into the design of the study and the planned analysis and will provide further input into the interpretation of research outputs, and to identify and prioritise future goals.

The second strand is broader engagement with research using electronic health records that is being undertaken as part of OpenSAFELY. This includes formation of an Oversight Board (which reviewed OpenPROMPT), publishing blogs explaining OpenSAFELY, presenting results of studies at public and professional events, soliciting feedback on studies via Twitter and email, conducting focus groups. As part of this project, the OpenPROMPT team reviewed existing qualitative studies on living with long COVID and conducted focus groups to explore the goals for the research and the feedback that patients want.

At study close, a summary will be published on the OpenPROMPT website^22^ and the study team will give an online interactive webinar describing the results and findings.

## Ethics and dissemination

### Safety reporting

As this is an observational study, an adverse event in this protocol is defined as any untoward medical occurrence in a patient that has occurred from research interventions only. In this case, the research interventions are questionnaires, and the risk to participants is minimal.

### Ethical review

The Study Coordination Centre has obtained approval from the LSHTM Research Ethics Committee (ref 28030), as well as a favourable opinion from the South Central—Berkshire B Research Ethics Committee (ref 22/SC/0198). Substantial amendments to the study protocol will obtain a favourable opinion by the local REC before they are implemented.

### Dissemination plan

All publications and presentations relating to the study will be authorised by the study management group and also follow the OpenSAFELY publication policy.^23^ The first publication of the study’s results will be in the name of the study management group, if this does not conflict with the journal’s policy. If there are named authors, these will include at least the trial’s chief investigator, statistician and study coordinator. Members of the study management group will be listed and contributors will be cited by name if published in a journal where this does not conflict with the journal’s policy. Authorship of parallel studies initiated outside of the study management group will be according to the individuals involved in the project but must acknowledge the contribution of the study management group and the study coordination centre.

All publications resulting from the OpenPROMPT study will be open access. Preprints will be made available prior to peer review.

### Data sharing

An aggregated version of the data will be included in publications arising from the study. The questionnaire data provided by participants will be made available to allow other researchers to benefit from this work in the future, and for a range of different studies and purposes. Opting-in to the study will require an affirmative response to agree to use of data in this way.

After completion of the study, a pseudonymised copy of the data will be held according to NHS England retention policy.^24^ Data access will be governed by LSHTM and will require researchers to complete a data access form. This will be explained in all publications arising from the study.

All other study documentation will be stored for a minimum of 5 years after the follow-up period.

## Supplementary Material

Reviewer comments

Author's manuscript
