# Supplementary material for: Protocol for an OpenSAFELY cohort study collecting patient-reported outcome measures using the TPP Airmid smartphone application and linked big data to quantify the health and economic costs of long COVID (OpenPROMPT)
Source: BMJ Open. 2023 Feb 17;13(2):e071261. doi: 10.1136/bmjopen-2022-071261 (PMC9943695; doi:10.1136/bmjopen-2022-071261)
Supplement: Author's manuscript [file bmjopen-2022-071261.draft_revisions.pdf]

BMJ Open

BMJ Open is committed to open peer review. As part of this commitment we make the peer review history of every article we publish publicly available.

When an article is published we post the peer reviewers' comments and the authors' responses online. We also post the versions of the paper that were used during peer review. These are the versions that the peer review comments apply to.

The versions of the paper that follow are the versions that were submitted during the peer review process. They are not the versions of record or the final published versions. They should not be cited or distributed as the published version of this manuscript.

BMJ Open is an open access journal and the full, final, typeset and author-corrected version of record of the manuscript is available on our site with no access controls, subscription charges or pay-per-view fees (http://bmjopen.bmj.com).

If you have any questions on BMJ Open's open peer review process please email info.bmjopen@bmj.com

BMJ Open

Protocol for an OpenSAFELY cohort study collecting Patient Reported Outcome Measures using the TPP Airmid smartphone application and linked big data to quantify the health and economic costs of long COVID (OpenPROMPT)

Journal:	*BMJ Open*
Manuscript ID	bmjopen-2022-071261
Article Type:	Protocol
Date Submitted by the Author:	31-Dec-2022

Complete List of Authors:	Herrett, Emily; London School of Hygiene and Tropical Medicine Department of Non-communicable Disease Epidemiology<br>Tomlin, Keith; London School of Hygiene and Tropical Medicine<br>Lin, Liang-Yu; London School of Hygiene and Tropical Medicine Faculty of Epidemiology and Population Health, Department of Non-communicable Disease Epidemiology<br>Tomlinson, Laurie; London School of Hygiene and Tropical Medicine, Epidemiology and Population Health<br>Jit, Mark; London School of Hygiene and Tropical Medicine, Epidemiology and Population Health<br>Briggs, Andrew ; London School of Hygiene & Tropical Medicine,<br>Marks, Michael; London School of Hygiene and Tropical Medicine, Clinical Research Department; Hospital for Tropical Diseases,<br>Sandmann, Frank; London School of Hygiene and Tropical Medicine Faculty of Epidemiology and Population Health<br>Parry, John; The Phoenix Partnership Leeds Ltd<br>Bates, Christopher; The Phoenix Partnership Leeds Ltd<br>Morley, Jessica; University of Oxford, Nuffield Department of Primary Care Health Sciences; University of Oxford, Oxford Internet Institute<br>Bacon, Seb; University of Oxford, Nuffield Department of Primary Care Health Sciences<br>Butler-Cole, Benjamin; University of Oxford Nuffield Department of Primary Care Health Sciences<br>Mahalingasivam, Viyaasan; London School of Hygiene and Tropical Medicine Faculty of Epidemiology and Population Health<br>Dennison, Alan; Patient and Public Involvement Steering Committee<br>Smith, Deb; University of Birmingham, Institute of Applied Health Research<br>Gabriel, Ethan; Patient and Public Involvement Steering Committee<br>Mehrkar, Amir; University of Oxford Nuffield Department of Primary Care Health Sciences<br>Goldacre, Ben; University of Oxford, Primary Care Health Sciences<br>Smeeth, Liam; London School of Hygiene and Tropical Medicine, Epidemiology and Population Health<br>Eggo, Rosalind M; London School of Hygiene and Tropical Medicine, Department of Infectious Disease Epidemiology

Keywords:	COVID-19, HEALTH ECONOMICS, Quality of Life

For peer review only - http://bmjopen.bmj.com/site/about/guidelines.xhtml

Protocol for an OpenSAFELY cohort study collecting Patient Reported Outcome Measures using the TPP Airmid smartphone application and linked big data to quantify the health and economic costs of long COVID (OpenPROMPT)

Dr Emily Herrett (corresponding author) (emily.herrett@lshtm.ac.uk)[1]
Mr Keith Tomlin (keith.tomlin@lshtm.ac.uk) [1]
Dr Liang-Yu Lin (liang-yu.lin@lshtm.ac.uk) [1]
Dr Laurie Tomlinson (laurie.tomlinson@lshtm.ac.uk) [1]
Professor Mark Jit (Mark.Jit@lshtm.ac.uk) [1]
Professor Andrew Briggs (Andrew.Briggs@lshtm.ac.uk) [1]
Dr Michael Marks (Michael.Marks@lshtm.ac.uk) [1,2]
Dr Frank Sandmann (Frank.Sandmann1@lshtm.ac.uk) [1]
Dr John Parry (john.parry@tpp-uk.com)[3]
Dr Chris Bates (chris.bates@tpp-uk.com) [3]
Jessica Morley (jessica.morley@phc.ox.ac.uk)[4]
Sebastian Bacon (sebastian.bacon@phc.ox.ac.uk) [4]
Benjamin Butler-Cole (ben.butler-cole@thedatalab.org) [4]
Dr Viyaasan Mahalingasivam (Viyaasan.Mahalingasivam1@lshtm.ac.uk) [1]
Alan Dennison (alan260760@aol.com)[5]
Debs Smith (debesmith1970@gmail.com) [5]
Ethan Gabriel (ethangabriel005@gmail.com) [5]
Dr Amir Mehrkar (amir.mehrkar@phc.ox.ac.uk) [4]
Professor Ben Goldacre (ben.goldacre@phc.ox.ac.uk) [4]
Professor Liam Smeeth (liam.smeeth@lshtm.ac.uk) [1]
Dr Rosalind Eggo (r.eggo@lshtm.ac.uk) [1]

1 London School of Hygiene and Tropical Medicine, London, UK

2 Hospital for Tropical Diseases, London, UK

3 The Phoenix Partnership, Leeds, UK

4 Nuffield Department of Primary Care Health Sciences, University of Oxford, Oxford, UK

5 Patient and Public Involvement Steering Committee, London, UK

For peer review only

Abstract

Introduction: The impact of long COVID on health-related quality-of-life and productivity is not currently known. It is important to understand who is worst affected by long COVID and the cost to the NHS and society, so that strategies like booster vaccines can be prioritised to the right people. OpenPROMPT aims to understand the impact of long COVID on health-related quality-of-life (HRQoL) in adults attending English primary care.

Methods and analysis: We will ask people to participate in the study through a smartphone app (Airmid), and completing a series of questionnaires held within the app. Questionnaires will ask about HRQoL, productivity and symptoms of long COVID. Participants will be asked to fill in the questionnaires once a month, for 90 days. Questionnaire responses will be linked, where possible, to participants' existing health records from primary care, secondary care, and COVID testing and vaccination data. Analysis will take place using the OpenSAFELY data platform and will estimate the impact of long COVID on HRQoL, productivity and cost to the NHS.

Ethics and dissemination: The Proportionate Review Sub-Committee of the South Central - Berkshire B Research Ethics Committee has also reviewed the study and have agreed that we can ask people to take part (22/SC/0198). Our results will provide information to support long-term care, and make recommendations for prevention of long COVID in the future.

Registration details: Clinicaltrials.gov NCT05552612

For peer review only

Strengths and limitations of this study

- OpenPROMPT will recruit people through a free app that is already in use within the NHS, meaning that the results will be stored in the safest possible way through NHS accredited data centres. Analysis will be through OpenSAFELY, which - as a Trusted Research Environment - will ensure participant confidentiality.
- participants are able to self assert their identity through the NHS app which links to the Airmid app, ensuring patient identity without imposing on care providers.
- OpenPROMPT will collect patient-reported outcome measures and link to the electronic health record, so is able to explore clinical and demographic factors related to long COVID, and explore healthcare utilisation across primary and secondary care.
- The advantage of OpenPROMPT is that it collects information directly from participants and links to the rich routine data held in the electronic health record.
- An important limitation is that participants in the study will be self-selected, which means their experience of long COVID may not be representative of all people with long COVID.

For peer review only

Introduction

Long COVID in the UK

There is growing evidence about the sequelae of COVID-19 infection.[1] Long COVID, or 'post-COVID syndrome' includes any symptoms after 12 weeks of infection not explained by another diagnosis.[2] It is anticipated that long COVID will represent a large part of the ultimate impact of the COVID-19 pandemic, both in terms of its long-lasting health effects[3] and economic costs generated through impacts on health-related quality-of-life (HRQoL), productivity[4] , especially on working-age adults who are ill or caring for those who are ill, and increased use of healthcare resources.[5] However, the size of this burden has not been comprehensively quantified.

In November 2022, persistent symptoms at least twelve weeks after infection are estimated to affect at least 2.1 million people in the UK (3.3% of the population).[6]

Mitigating the impact of long COVID

To mitigate the impact of long COVID, it is vital to collect information that will enable the NHS to make healthcare decisions, evaluate and prioritise vaccination boosters and other COVID-related interventions. Firstly, interventions used by the NHS are evaluated for cost-effectiveness by the National Institute for Health and Care Excellence (NICE). This requires quantitative information about impacts measured using (i) standardised measures of changes in patient HRQoL (including physical and mental health effects) using the EuroQoL EQ-5D instrument, and (ii) formal quantification of the healthcare burden of the condition and its treatment. In addition, evaluations by other organisations often also require (iii) measures of society-level impacts such as decreases in productivity in people with the condition. Secondly, patient-reported outcome measures such as HRQoL are vital for monitoring the effectiveness of interventions funded by the NHS, as well as to guide clinical decision-making.

Gaps in the evidence

For peer review only - http://bmjopen.bmj.com/site/about/guidelines.xhtml

There is growing evidence about the health economic impact of COVID and its sequelae, with lower quality of life scores reported among those with COVID, post COVID syndrome or long COVID, compared to those without COVID or without ongoing symptoms.[7–9]

However, a comprehensive analysis of HRQoL in long COVID should take account of changes in the circulating strain of COVID, the impact of vaccination, time after COVID, and should compare those with long COVID to those not experiencing ongoing symptoms. To date, the existing literature has not explored HRQoL using the validated EQ-5D instrument among those living with long COVID in England in such depth. This means that the impact of long COVID cannot be reliably included in future planning for COVID-19 epidemics, nor can we accurately measure the true scale of its effects or burden on the NHS, or the cost-effectiveness of interventions to mitigate these effects. Determining how long COVID affects HRQoL and NHS costs is critical for evaluation and for planning the future of COVID-19 response in the UK. This is particularly true in light of some evidence suggesting a reduced risk in vaccinated individuals since the emergence of the Omicron strain of SARS-CoV-2,[10] while the risk of long COVID increases with the number of (re-)infections.[11] Lastly, with a focus on long-term disability in people living with long COVID, the study is also responding directly to the research needs identified by WHO in October 2022.[7]

To address these gaps in the evidence, this study will use novel methods to assess the impact of long COVID on HRQoL, healthcare utilization, cost to the NHS and productivity among both hospitalised and non-hospitalised COVID cases, taking account of the timing of COVID diagnosis, and the number and timing of infections and vaccinations. We will also investigate the effect of demographic and clinical factors, and perform a health economic evaluation of future vaccination strategies, incorporating the impact on long COVID.

Methods and analysis

Study design and setting

OpenPROMPT is a cohort study among adults in England, recruiting between November 2022 and May 2023. This study is being conducted in collaboration with TPP, a GP software

For peer review only - http://bmjopen.bmj.com/site/about/guidelines.xhtml

provider. TPP's software system is called SystmOne and holds patient electronic health records for 40% of the population of England. Their in-house smartphone app, Airmid,[12] has been developed as a patient-facing mobile application, and links directly to SystmOne records. It was created to allow appointment booking, for patients to access their own medical records, and for patients to input their own data to feed into their medical record. Airmid's Research Module can run surveys and bespoke questionnaires, with results recorded in a patient's Personal Health Record. Airmid requires either a NHS Login or TPP SystmOnline Username and Password. Any individual can download the Airmid app, even if their GP does not use TPP software. Airmid and SystmOne are being provided by TPP for no charge.

Inclusion criteria

Any adult in England can take part in the study (irrespective of long COVID/COVID status), if they are able to read and understand English, and download and use the Airmid app (available on iOS and Android free of charge).

Recruitment

For people who already have the app installed on their device, the study page will be visible when they log in to the app, and they will be able to enrol in the study. The study will also be advertised to people registered at general practices that use SystmOne software. GPs at these practices will send text messages or emails to all adults in the system (who they have permission to contact). The text message or email will contain a link to the study website. This will hold information on the study and a guide to participation, through the Airmid app. The guide will include a link to instructions on how to download the Airmid app and where to find the study within the app. The study will be advertised more broadly through social media platforms to long COVID groups and the general population. Potential participants will be invited to download the app and opt-in to the study.

Potential participants will be asked to read the Patient Information Sheet and consent statements within the app, and will move an 'opt-in' slider within the app to provide an affirmative response that they have read and understood the information, and agree to the

For peer review only - http://bmjopen.bmj.com/site/about/guidelines.xhtml

consent statements. Consent will be recorded and dated within the participant's Personal Health Record.

We will monitor recruitment weekly and compare against our targets for recruitment. We will repeat efforts to increase recruitment through social media campaigns in the first week of each month during the study period.

Study questionnaires

The study questionnaires will be available within the Airmid app once a participant has opted-in to the study. There are two study-specific questionnaires, enquiring about participants' demographic characteristics, and about their experience of COVID, Long COVID and COVID vaccination. The study also asks participants to complete four validated questionnaires: (1) EuroQoL EQ-5D 5L,[13] measuring HRQoL and enabling calculation of quality-adjusted life years lost due to acute COVID and long COVID; (2) the Work Productivity and Activity Impairment Questionnaire: Specific Health Problem 2.0, measuring productivity and estimating the economic value of productivity loss in people with long COVID;[14] (3) the MRC Dyspnoea scale measuring breathlessness,[15] and (4) the FACIT tool, measuring fatigue.[16] These symptom questionnaires will allow valid symptom comparisons between those with and without long COVID.

Data linkage

For participants who are registered at a general practice that uses TPP SystmOne software (around 40% of English general practices),[17] OpenPROMPT will link participant questionnaire data to the OpenSAFELY-TPP platform: initially to enhance the existing OpenSAFELY-TPP data with results of questionnaires, then to analyse individual-level data and translate the impacts to the population level. OpenSAFELY-TPP is a secure platform for analysis of electronic patient health records and is a Trusted Research Environment with strict governance to maximise patient confidentiality and privacy. The main purpose of OpenSAFELY-TPP is to conduct research in the public interest to deepen our understanding of the Covid-19 pandemic. OpenSAFELY-TPP will incorporate the questionnaire data into the existing OpenSAFELY framework. The data will be linked to existing data and

For peer review only

infrastructure via strongly pseudonymised NHS number, based on industry standard encryption. Access to data within the OpenSAFELY TPP platform is under the COVID-19 Control of Patient Information (COPI) Regulation 3. This means that data can be processed within OpenSAFELY, without the need for each GP practice to consent. Additionally, OpenPROMPT will seek informed patient-level consent for analysis of study-specific data, with the aim that after the withdrawal of COPI the study will remain accessible.

[INSERT FIGURE 1]

OpenSAFELY-TPP holds linked data including primary care, COVID testing, hospitalisations, COVID-19 Blueteq treatment data and mortality data. Primary care data from OpenSAFELY will link to coded consultations with the general practice team, diagnosis codes, prescriptions, referral codes and tests undertaken in primary care.

For participants who are not registered at a practice using TPP SystmOne software, the study data will be limited to the questionnaire results, and analysis will take place outside the OpenSAFELY environment.

Follow-up

Participants will be prompted by a push notification (a pop up on their phone screen) to respond to each questionnaire at the time of recruitment, and then at 30, 60 and 90 days after recruitment. The demographic questionnaire will be asked only at baseline (including for example, age, sex, relationship status, socioeconomic status, etc). Participants will be invited to complete all questionnaires, but partially complete data will also be available for analysis.

System reports will flag incomplete questionnaires and one additional push notification reminder will be sent to participants who have not completed any questionnaires within 5 days of the initial notification. Active follow-up will end at 90 days when participants are

For peer review only - http://bmjopen.bmj.com/site/about/guidelines.xhtml

asked to complete their final set of questionnaires. Ongoing, passive follow-up will be continued within the routinely collected electronic health record once the data linkage is in place. At the point of recruitment, participants will be offered the opportunity to consent to linkage and use of their data by researchers.

Participants are free to withdraw from the study at any time and there will be no implications for their care. Withdrawal of consent will occur through Airmid, by moving the opt-in toggle to opt-out.

Study size

The primary research question is to estimate the impact of long COVID on HRQoL as measured using EQ-5D scores at multiple timepoints. Average quality of life in a study of people with severe hospitalised COVID estimated *pre-COVID* HRQoL scores (EQ-5D) to be 0.9655 (standard deviation 0.0958)) and then 0.7054 (standard deviation 0.2514) six months after COVID.[18] We anticipate that people with long COVID participating in the present study will be a mixture of hospitalised and non-hospitalised patients, so are likely to be affected to a lesser extent than a cohort of only hospitalised patients. A second study in a general population cohort estimated EQ-5D scores to be 0.979 (SD 0.0530) among people with no chronic diseases, and 0.9360 (SD 0.112) among those with one chronic disease.[19]

We have therefore performed a power calculation based on the conservative estimates of 0.979 in participants without long COVID, and 0.9360 in those with long COVID (comparable to people with one chronic disease). At the time of calculation, population prevalence of long COVID was estimated to be 2.7%. We assumed that we will over-recruit people with long COVID, due to our focused recruitment strategy. Assuming that 5% of our participants have long COVID, this gives a sample size of 1172 participants to achieve 80% power (alpha 0.05) (corresponding to 1116 in the group without long COVID and 56 among those with long COVID). We intend to keep recruiting beyond this target, as there is no additional cost to the study and minimal risk to the additional respondents, and it will increase our power to address future research questions.

For peer review only - http://bmjopen.bmj.com/site/about/guidelines.xhtml

To explore sampling bias, we will compare characteristics of people registered at GPs using TPP SysmOne software who take part in the study to those registered at other practices, using data from their electronic health record, and additionally compare our results to those of the Office for National Statistics[20] and the Institute for Fiscal Studies[21]

Analysis plan

The main outcome will be QALYs lost after an episode of COVID, calculated from HRQoL as measured by the EuroQOL EQ-5D questionnaire at multiple timepoints. OpenPROMPT will also explore outcomes based on the results from the symptom and productivity questionnaire results.

The main exposure will be long COVID. This will be self-reported by participants in the questionnaires, and recorded in routinely collected data in OpenSAFELY (primary and secondary care data and COVID testing data). The definition of long COVID used in this study will be based on a combination of self-report, diagnoses and referrals for long COVID; the definition will be explored with sensitivity analyses

OpenPROMPT will analyse individual-level determinants of HRQoL deficits in people with long COVID, including key variables such as ethnicity, age, socioeconomic status, and pre-existing health conditions.

This will generate a strong health economic evidence base for assessing the impact of long COVID and evaluating future interventions (including essential inputs to cost-effectiveness analyses of interventions like vaccination boosters, therapeutics and non-pharmaceutical interventions), and demonstrate the effect of long COVID on the health system so far.

Patient and public involvement

We have two streams of patients and public involvement. First, OpenPROMPT recruited a three person steering group, including members with long COVID, those experienced in patient and public involvement or with an interest in long COVID. The steering group aims

to meet with the research team online every 6 months. This group has given input into the design of the study and the planned analysis and will provide further input into the interpretation of research outputs, and to identify and prioritise future goals.

The second strand is broader engagement with research using electronic health records that is being undertaken as part of OpenSAFELY. This includes formation of an Oversight Board (which reviewed OpenPROMPT), publishing blogs explaining OpenSAFELY, presenting results of studies at public and professional events, soliciting feedback on studies via Twitter and email, conducting focus groups. As part of this project, the OpenPROMPT team reviewed existing qualitative studies on living with long COVID and conducted focus groups to explore the goals for the research and the feedback that patients want.

At study close, a summary will be published on the OpenPROMPT website[22] and the study team will give an online interactive webinar describing the results and findings.

Ethics and dissemination

Safety reporting

As this is an observational study, an adverse event in this protocol is defined as any untoward medical occurrence in a patient that has occurred from research interventions only. In this case the research interventions are questionnaires, and the risk to participants is minimal.

Ethical review

The Study Coordination Centre has obtained approval from the LSHTM Research Ethics Committee (ref 28030), as well as a favourable opinion from the South Central – Berkshire B Research Ethics Committee (ref 22/SC/0198). Substantial amendments to the study protocol will obtain a favourable opinion by the local REC before they are implemented.

Dissemination plan

All publications and presentations relating to the study will be authorised by the Study Management Group and also follow the OpenSAFELY publication policy.[23] The first

For peer review only - http://bmjopen.bmj.com/site/about/guidelines.xhtml

publication of the study's results will be in the name of the Study Management Group, if this does not conflict with the journal's policy. If there are named authors, these will include at least the trial's Chief Investigator, Statistician and Study Coordinator. Members of the Study Management Group will be listed and contributors will be cited by name if published in a journal where this does not conflict with the journal's policy. Authorship of parallel studies initiated outside of the Study Management Group will be according to the individuals involved in the project but must acknowledge the contribution of the Study Management Group and the Study Coordination Centre.

All publications resulting from the OpenPROMPT study will be open access. Pre-prints will be made available prior to peer review.

Data sharing

An aggregated version of the data will be included in publications arising from the study. The questionnaire data provided by participants will be made available to allow other researchers to benefit from this work in the future, and for a range of different studies and purposes. Opting-in to the study will require an affirmative response to agree to use of data in this way.

After completion of the study, a pseudonymised copy of the data will be held according to NHS England retention policy.[24] Data access will be governed by LSHTM and will require researchers to complete a data access form. This will be explained in all publications arising from the study.

All other study documentation will be stored for a minimum of five years after the follow-up period.

Authors' contributions

EH, RE, KT, and LYL drafted the protocol and all other authors reviewed.

For peer review only - http://bmjopen.bmj.com/site/about/guidelines.xhtml

Competing interests statement

No competing interests are declared.

Funding statement

This work was supported by the NIHR Long COVID grant number COV-LT2-0073. The views expressed are those of the authors and not necessarily those of the NIHR or the Department of Health and Social Care.

For peer review only

References

1. Franco, J. V. A. *et al.* Long-Term Health Symptoms and Sequelae Following SARS-CoV-2 Infection: An Evidence Map. *Int J Environ Res Public Health* **19**, 9915 (2022).
2. Overview | COVID-19 rapid guideline: managing the long-term effects of COVID-19 | Guidance | NICE. https://www.nice.org.uk/guidance/ng188.
3. Al-Aly, Z., Xie, Y. & Bowe, B. High-dimensional characterization of post-acute sequelae of COVID-19. *Nature* **594**, 259–264 (2021).
4. Jacobson, K. B. *et al.* Patients With Uncomplicated Coronavirus Disease 2019 (COVID-19) Have Long-Term Persistent Symptoms and Functional Impairment Similar to Patients with Severe COVID-19: A Cautionary Tale During a Global Pandemic. *Clin Infect Dis* **73**, e826–e829 (2021).
5. Whittaker, H. *et al.* Post-Acute COVID-19 Sequelae in Cases Managed in the Community or Hospital in the UK: A Population Based Stud. Preprint at https://doi.org/10.2139/ssrn.3820510 (2021).
6. Prevalence of ongoing symptoms following coronavirus (COVID-19) infection in the UK - Office for National Statistics.

For peer review only - http://bmjopen.bmj.com/site/about/guidelines.xhtml

https://www.ons.gov.uk/peoplepopulationandcommunity/healthandsocialcare/conditionsanddiseases/bulletins/prevalenceofongoingsymptomsfollowingcoronaviruscovid19infectionintheuk/3november2022.

7. de Oliveira Almeida, K. *et al.* A systematic review on physical function, activities of daily living and health-related quality of life in COVID-19 survivors. *Chronic Illn* 17423953221089308 (2022) doi:10.1177/17423953221089309.
8. Malik, P. *et al.* Post-acute COVID-19 syndrome (PCS) and health-related quality of life (HRQoL)-A systematic review and meta-analysis. *J Med Virol* **94**, 253–262 (2022).
9. Nandasena, H. M. R. K. G., Pathirathna, M. L., Atapattu, A. M. M. P. & Prasanga, P. T. S. Quality of life of COVID 19 patients after discharge: Systematic review. *PLOS ONE* **17**, e0263941 (2022).
10. Antonelli, M., Pujol, J. C., Spector, T. D., Ourselin, S. & Steves, C. J. Risk of long COVID associated with delta versus omicron variants of SARS-CoV-2. *The Lancet* **399**, 2263–2264 (2022).
11. Bowe, B., Xie, Y. & Al-Aly, Z. Acute and postacute sequelae associated with SARS-CoV-2 reinfection. *Nat Med* **28**, 2398–2405 (2022).
12. Airmid. *TPP* https://tpp-uk.com/products/airmid/ (2020).
13. EQ-5D. https://euroqol.org/.
14. Reilly, M. Work Productivity and Activity Impairment Questionnaire: 2.
15. MRC Dyspnoea Scale | Primary Care Respiratory Society. https://www.pcrs-uk.org/mrc-dyspnoea-scale.
16. FACIT-F. *FACIT Group* https://www.facit.org/measures/FACIT-F.
17. OpenSAFELY: Representativeness of ... | Wellcome Open Research. https://wellcomeopenresearch.org/articles/7-191/v1.

For peer review only - http://bmjopen.bmj.com/site/about/guidelines.xhtml

18. Taboada, M. *et al.* Quality of life, functional status, and persistent symptoms after intensive care of COVID-19 patients. *Br J Anaesth* **126**, e110–e113 (2021).

19. Ping, W. *et al.* Evaluation of health-related quality of life using EQ-5D in China during the COVID-19 pandemic. *PLOS ONE* **15**, e0234850 (2020).

20. Prevalence of ongoing symptoms following coronavirus (COVID-19) infection in the UK - Office for National Statistics. https://www.ons.gov.uk/peoplepopulationandcommunity/healthandsocialcare/conditionsanddiseases/bulletins/prevalenceofongoingsymptomsfollowingcoronaviruscovid19infectionintheuk/7april2022.

21. Long COVID and the labour market. *Institute for Fiscal Studies* https://ifs.org.uk/publications/long-covid-and-labour-market.

22. OpenSAFELY long COVID study website. https://www.opensafely.org/studies/long-covid/.

23. OpenSAFELY: Policies for Researchers. https://www.opensafely.org/policies-for-researchers/.

24. NHS England » OpenSAFELY – the Coronavirus (COVID-19) Research Platform. https://www.england.nhs.uk/contact-us/privacy-notice/how-we-use-your-information/covid-19-response/coronavirus-covid-19-research-platform/.

Word Count
2749

Figure legends

Figure 1. Overview of study

For peer review only

For peer review only

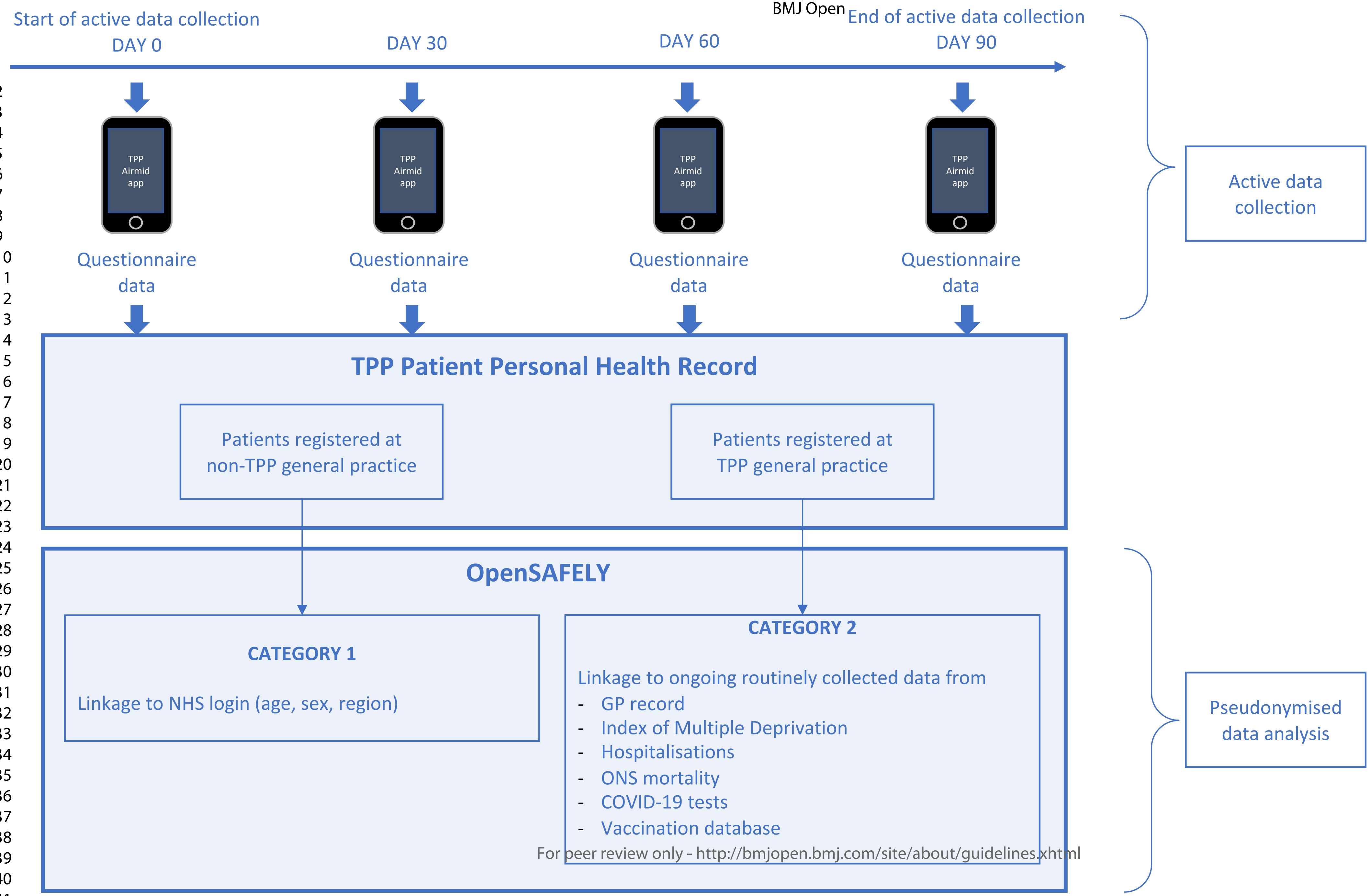

For peer review only - http://bmjopen.bmj.com/site/about/guidelines.xhtml

For peer review only - http://bmjopen.bmj.com/site/about/guidelines.xhtml

Reporting checklist for cohort study.

Based on the STROBE cohort guidelines.

Instructions to authors

Complete this checklist by entering the page numbers from your manuscript where readers will find each of the items listed below.

Your article may not currently address all the items on the checklist. Please modify your text to include the missing information. If you are certain that an item does not apply, please write "n/a" and provide a short explanation.

Upload your completed checklist as an extra file when you submit to a journal.

In your methods section, say that you used the STROBE cohortreporting guidelines, and cite them as:

von Elm E, Altman DG, Egger M, Pocock SJ, Gotzsche PC, Vandenbroucke JP. The Strengthening the Reporting of Observational Studies in Epidemiology (STROBE) Statement: guidelines for reporting observational studies.

		Reporting Item	Page Number
**Title and abstract**			
Title	#1a	Indicate the study's design with a commonly used term in the title or the abstract	1
Abstract	#1b	Provide in the abstract an informative and balanced summary of what was done and what was found	2
**Introduction**			
Background / rationale	#2	Explain the scientific background and rationale for the investigation being reported	4
Objectives	#3	State specific objectives, including any prespecified hypotheses	5
**Methods**			
Study design	#4	Present key elements of study design early in the paper	5-6

For peer review only

Setting	#5	Describe the setting, locations, and relevant dates, including periods of recruitment, exposure, follow-up, and data collection	5-6
Eligibility criteria	#6a	Give the eligibility criteria, and the sources and methods of selection of participants. Describe methods of follow-up.	6
Eligibility criteria	#6b	For matched studies, give matching criteria and number of exposed and unexposed	n/a
Variables	#7	Clearly define all outcomes, exposures, predictors, potential confounders, and effect modifiers. Give diagnostic criteria, if applicable	7 - questionnaires
Data sources / measurement	#8	For each variable of interest give sources of data and details of methods of assessment (measurement). Describe comparability of assessment methods if there is more than one group. Give information separately for for exposed and unexposed groups if applicable.	7
Bias	#9	Describe any efforts to address potential sources of bias	10
Study size	#10	Explain how the study size was arrived at	9-10
Quantitative variables	#11	Explain how quantitative variables were handled in the analyses. If applicable, describe which groupings were chosen, and why	n/a
Statistical methods	#12a	Describe all statistical methods, including those used to control for confounding	n/a - protocol
Statistical methods	#12b	Describe any methods used to examine subgroups and interactions	n/a protocol
Statistical methods	#12c	Explain how missing data were addressed	n/a - protocol
Statistical methods	#12d	If applicable, explain how loss to follow-up was addressed	n/a - protocol

Statistical methods	#12e	Describe any sensitivity analyses

For peer review only

		which confounders were adjusted for and why they were included	
Main results	#16b	Report category boundaries when continuous variables were categorized	n/a protocol
Main results	#16c	If relevant, consider translating estimates of relative risk into absolute risk for a meaningful time period	n/a protocol
Other analyses	#17	Report other analyses done—eg analyses of subgroups and interactions, and sensitivity analyses	n/a protocol
**Discussion**			
Key results	#18	Summarise key results with reference to study objectives	n/a protocol
Limitations	#19	Discuss limitations of the study, taking into account sources of potential bias or imprecision. Discuss both direction and magnitude of any potential bias.	n/a protocol
Interpretation	#20	Give a cautious overall interpretation considering objectives, limitations, multiplicity of analyses, results from similar studies, and other relevant evidence.	n/a protocol
Generalisability	#21	Discuss the generalisability (external validity) of the study results	n/a protocol
**Other Information**			
Funding	#22	Give the source of funding and the role of the funders for the present study and, if applicable, for the original study on which the present article is based	15

None The STROBE checklist is distributed under the terms of the Creative Commons Attribution License CC-BY. This checklist can be completed online using https://www.goodreports.org/, a tool made by the EQUATOR Network in collaboration with Penelope.ai

For peer review only

Protocol for an OpenSAFELY cohort study collecting Patient Reported Outcome Measures using the TPP Airmid smartphone application and linked big data to quantify the health and economic costs of long COVID (OpenPROMPT)

Journal:	*BMJ Open*
Manuscript ID	bmjopen-2022-071261.R1
Article Type:	Protocol
Date Submitted by the Author:	20-Jan-2023
Complete List of Authors:	Herrett, Emily; London School of Hygiene and Tropical Medicine Department of Non-communicable Disease Epidemiology<br>Tomlin, Keith; London School of Hygiene and Tropical Medicine<br>Lin, Liang-Yu; London School of Hygiene and Tropical Medicine Faculty of Epidemiology and Population Health, Department of Non-communicable Disease Epidemiology<br>Tomlinson, Laurie; London School of Hygiene and Tropical Medicine, Epidemiology and Population Health<br>Jit, Mark; London School of Hygiene and Tropical Medicine, Epidemiology and Population Health<br>Briggs, Andrew ; London School of Hygiene & Tropical Medicine,<br>Marks, Michael; London School of Hygiene and Tropical Medicine, Clinical Research Department; Hospital for Tropical Diseases,<br>Sandmann, Frank; London School of Hygiene and Tropical Medicine Faculty of Epidemiology and Population Health<br>Parry, John; TPP<br>Bates, Christopher; TPP<br>Morley, Jessica; University of Oxford, Nuffield Department of Primary Care Health Sciences; University of Oxford, Oxford Internet Institute<br>Bacon, Seb; University of Oxford, Nuffield Department of Primary Care Health Sciences<br>Butler-Cole, Benjamin; University of Oxford Nuffield Department of Primary Care Health Sciences<br>Mahalingasivam, Viyaasan; London School of Hygiene and Tropical Medicine Faculty of Epidemiology and Population Health<br>Dennison, Alan; Patient and Public Involvement Steering Committee<br>Smith, Deb; University of Birmingham, Institute of Applied Health Research<br>Gabriel, Ethan; Patient and Public Involvement Steering Committee<br>Mehrkar, Amir; University of Oxford Nuffield Department of Primary Care Health Sciences<br>Goldacre, Ben; University of Oxford, Primary Care Health Sciences<br>Smeeth, Liam; London School of Hygiene and Tropical Medicine, Epidemiology and Population Health<br>Eggo, Rosalind M; London School of Hygiene and Tropical Medicine, Department of Infectious Disease Epidemiology
Primary Subject	Health economics

Heading:	
Secondary Subject Heading:	Epidemiology
Keywords:	COVID-19, HEALTH ECONOMICS, Quality of Life

SCHOLARONE™
Manuscripts

Protocol for an OpenSAFELY cohort study collecting Patient Reported Outcome Measures using the TPP Airmid smartphone application and linked big data to quantify the health and economic costs of long COVID (OpenPROMPT)

Dr Emily Herrett (corresponding author) (emily.herrett@lshtm.ac.uk)[1]
Mr Keith Tomlin (keith.tomlin@lshtm.ac.uk) [1]
Dr Liang-Yu Lin (liang-yu.lin@lshtm.ac.uk) [1]
Dr Laurie Tomlinson (laurie.tomlinson@lshtm.ac.uk) [1]
Professor Mark Jit (Mark.Jit@lshtm.ac.uk) [1]
Professor Andrew Briggs (Andrew.Briggs@lshtm.ac.uk) [1]
Dr Michael Marks (Michael.Marks@lshtm.ac.uk) [1,2]
Dr Frank Sandmann (Frank.Sandmann1@lshtm.ac.uk) [1]
Dr John Parry (john.parry@tpp-uk.com)[3]
Dr Chris Bates (chris.bates@tpp-uk.com) [3]
Jessica Morley (jessica.morley@phc.ox.ac.uk)[4]
Sebastian Bacon (sebastian.bacon@phc.ox.ac.uk) [4]
Benjamin Butler-Cole (ben.butler-cole@thedatalab.org) [4]
Dr Viyaasan Mahalingasivam (Viyaasan.Mahalingasivam1@lshtm.ac.uk) [1]
Alan Dennison (alan260760@aol.com)[5]
Deb Smith (debesmith1970@gmail.com) [5]
Ethan Gabriel (ethangabriel005@gmail.com) [5]
Dr Amir Mehrkar (amir.mehrkar@phc.ox.ac.uk) [4]
Professor Ben Goldacre (ben.goldacre@phc.ox.ac.uk) [4]
Professor Liam Smeeth (liam.smeeth@lshtm.ac.uk) [1]
Dr Rosalind Eggo (r.eggo@lshtm.ac.uk) [1]

1 London School of Hygiene and Tropical Medicine, London, UK

2 Hospital for Tropical Diseases, London, UK

3 TPP, Leeds, UK

4 Nuffield Department of Primary Care Health Sciences, University of Oxford, Oxford, UK

5 Patient and Public Involvement Steering Committee, London, UK

For peer review only - http://bmjopen.bmj.com/site/about/guidelines.xhtml

Abstract

Introduction: The impact of long COVID on health-related quality-of-life and productivity is not currently known. It is important to understand who is worst affected by long COVID and the cost to the NHS and society, so that strategies like booster vaccines can be prioritised to the right people. OpenPROMPT aims to understand the impact of long COVID on health-related quality-of-life (HRQoL) in adults attending English primary care.

Methods and analysis: We will ask people to participate in this cohort study through a smartphone app (Airmid), and completing a series of questionnaires held within the app. Questionnaires will ask about HRQoL, productivity and symptoms of long COVID. Participants will be asked to fill in the questionnaires once a month, for 90 days. Questionnaire responses will be linked, where possible, to participants' existing health records from primary care, secondary care, and COVID testing and vaccination data. Analysis will take place using the OpenSAFELY data platform and will estimate the impact of long COVID on HRQoL, productivity and cost to the NHS.

Ethics and dissemination: The Proportionate Review Sub-Committee of the South Central - Berkshire B Research Ethics Committee has also reviewed and approved the study and have agreed that we can ask people to take part (22/SC/0198). Our results will provide information to support long-term care, and make recommendations for prevention of long COVID in the future.

Registration details: Clinicaltrials.gov NCT05552612

For peer review only

Strengths and limitations of this study

- OpenPROMPT will recruit people through a free app that is already in use within the NHS, meaning that the results will be stored in the safest possible way through NHS accredited data centres.
- Analysis will be through OpenSAFELY, which - as a Trusted Research Environment - will ensure participant confidentiality.
- Participants are able to self assert their identity through the NHS app which links to the Airmid app, ensuring patient identity without imposing on care providers.
- The advantage of OpenPROMPT is that it collects information directly from participants and links to the rich routine data held in the electronic health record.
- An important limitation is that participants in the study will be self-selected, which means their experience of long COVID may not be representative of all people with long COVID.

For peer review only

Introduction

Long COVID in the UK

There is growing evidence about the sequelae of COVID-19 infection.[1] Long COVID, or 'post-COVID syndrome' includes any symptoms after 12 weeks of infection not explained by another diagnosis.[2] It is anticipated that long COVID will represent a large part of the ultimate impact of the COVID-19 pandemic, both in terms of its long-lasting health effects[3] and economic costs generated through impacts on health-related quality-of-life (HRQoL), productivity[4] , especially on working-age adults who are ill or caring for those who are ill, and increased use of healthcare resources.[5] However, the size of this burden has not been comprehensively quantified.

In November 2022, persistent symptoms at least twelve weeks after infection are estimated to affect at least 2.1 million people in the UK (3.3% of the population).[6]

Mitigating the impact of long COVID

To mitigate the impact of long COVID, it is vital to collect information that will enable the NHS to make healthcare decisions, evaluate and prioritise vaccination boosters and other COVID-related interventions. Firstly, interventions used by the NHS are evaluated for cost-effectiveness by the National Institute for Health and Care Excellence (NICE). This requires quantitative information about impacts measured using (i) standardised measures of changes in patient HRQoL (including physical and mental health effects) using the EuroQoL EQ-5D instrument, and (ii) formal quantification of the healthcare burden of the condition and its treatment. In addition, evaluations by other organisations often also require (iii) measures of society-level impacts such as decreases in productivity in people with the condition. Secondly, patient-reported outcome measures such as HRQoL are vital for monitoring the effectiveness of interventions funded by the NHS, as well as to guide clinical decision-making.

Gaps in the evidence

For peer review only - http://bmjopen.bmj.com/site/about/guidelines.xhtml

There is growing evidence about the health economic impact of COVID and its sequelae, with lower quality of life scores reported among those with COVID, post COVID syndrome or long COVID, compared to those without COVID or without ongoing symptoms.[7–9]

However, a comprehensive analysis of HRQoL in long COVID should take account of changes in the circulating strain of COVID, the impact of vaccination, time after COVID, and should compare those with long COVID to those not experiencing ongoing symptoms. To date, the existing literature has not explored HRQoL using the validated EQ-5D instrument among those living with long COVID in England in such depth. This means that the impact of long COVID cannot be reliably included in future planning for COVID-19 epidemics, nor can we accurately measure the true scale of its effects or burden on the NHS, or the cost-effectiveness of interventions to mitigate these effects. Determining how long COVID affects HRQoL and NHS costs is critical for evaluation and for planning the future of COVID-19 response in the UK. This is particularly true in light of some evidence suggesting a reduced risk in vaccinated individuals since the emergence of the Omicron strain of SARS-CoV-2,[10] while the risk of long COVID increases with the number of (re-)infections.[11] Lastly, with a focus on long-term disability in people living with long COVID, the study is also responding directly to the research needs identified by WHO in October 2022.[7]

To address these gaps in the evidence, this study will use novel methods to assess the impact of long COVID on HRQoL, healthcare utilization, cost to the NHS and productivity among both hospitalised and non-hospitalised COVID cases, taking account of the timing of COVID diagnosis, and the number and timing of infections and vaccinations. We will also investigate the effect of demographic and clinical factors, and perform a health economic evaluation of future vaccination strategies, incorporating the impact on long COVID.

Methods and analysis

Study design and setting

OpenPROMPT is a cohort study among adults in England, recruiting between November 2022 and May 2023. This study is being conducted in collaboration with TPP, a GP software

For peer review only - http://bmjopen.bmj.com/site/about/guidelines.xhtml

provider. TPP's software system is called SystmOne and holds patient electronic health records for 40% of the population of England. Their in-house smartphone app, Airmid,[12] has been developed as a patient-facing mobile application, and links directly to SystmOne records. It was created to allow appointment booking, for patients to access their own medical records, and for patients to input their own data to feed into their medical record. Airmid's Research Module can run surveys and bespoke questionnaires, with results recorded in a patient's Personal Health Record. Airmid requires either a NHS Login or TPP SystmOnline Username and Password. Any individual can download the Airmid app, even if their GP does not use TPP software. Airmid and SystmOne are being provided by TPP for no charge.

Inclusion criteria

Any adult in England can take part in the study (irrespective of long COVID/COVID status), if they are able to read and understand English, and download and use the Airmid app (available on iOS and Android free of charge).

Recruitment

For people who already have the app installed on their device, the study page will be visible when they log in to the app, and they will be able to enrol in the study. The study will also be advertised to people registered at general practices that use SystmOne software. GPs at these practices will send text messages or emails to all adults in the system (who they have permission to contact). The text message or email will contain a link to the study website. This will hold information on the study and a guide to participation, through the Airmid app. The guide will include a link to instructions on how to download the Airmid app and where to find the study within the app. The study will be advertised more broadly through social media platforms to long COVID groups and the general population. Potential participants will be invited to download the app and opt-in to the study.

Potential participants will be asked to read the Patient Information Sheet and consent statements within the app, and will move an 'opt-in' slider within the app to provide an affirmative response that they have read and understood the information, and agree to the

For peer review only - http://bmjopen.bmj.com/site/about/guidelines.xhtml

consent statements. Consent will be recorded and dated within the participant's Personal Health Record.

We will monitor recruitment weekly and compare against our targets for recruitment. We will repeat efforts to increase recruitment through social media campaigns in the first week of each month during the study period.

Study questionnaires

The study questionnaires will be available within the Airmid app once a participant has opted-in to the study. There are two study-specific questionnaires, enquiring about participants' demographic characteristics, and about their experience of COVID, Long COVID and COVID vaccination. The study also asks participants to complete four validated questionnaires: (1) EuroQoL EQ-5D 5L,[13] measuring HRQoL and enabling calculation of quality-adjusted life years lost due to acute COVID and long COVID; (2) the Work Productivity and Activity Impairment Questionnaire: Specific Health Problem 2.0, measuring productivity and estimating the economic value of productivity loss in people with long COVID;[14] (3) the MRC Dyspnoea scale measuring breathlessness,[15] and (4) the FACIT tool, measuring fatigue.[16] These symptom questionnaires will allow valid symptom comparisons between those with and without long COVID.

Data linkage

For participants who are registered at a general practice that uses TPP SystmOne software (around 40% of English general practices),[17] OpenPROMPT will link participant questionnaire data to the OpenSAFELY-TPP platform: initially to enhance the existing OpenSAFELY-TPP data with results of questionnaires, then to analyse individual-level data and translate the impacts to the population level. OpenSAFELY-TPP is a secure platform for analysis of electronic patient health records and is a Trusted Research Environment with strict governance to maximise patient confidentiality and privacy. The main purpose of OpenSAFELY-TPP is to conduct research in the public interest to deepen our understanding of the Covid-19 pandemic. OpenSAFELY-TPP will incorporate the questionnaire data into the existing OpenSAFELY framework. The data will be linked to existing data and

For peer review only

infrastructure via strongly pseudonymised NHS number, based on industry standard encryption. Access to data within the OpenSAFELY TPP platform is under the COVID-19 Control of Patient Information (COPI) Regulation 3. This means that data can be processed within OpenSAFELY, without the need for each GP practice to consent. Additionally, OpenPROMPT will seek informed patient-level consent for analysis of study-specific data, with the aim that after the withdrawal of COPI the study will remain accessible.

[INSERT FIGURE 1]

For peer review only

OpenSAFELY-TPP holds linked data including primary care, COVID testing, hospitalisations, COVID-19 Blueteq treatment data and mortality data. Primary care data from OpenSAFELY will link to coded consultations with the general practice team, diagnosis codes, prescriptions, referral codes and tests undertaken in primary care.

For participants who are not registered at a practice using TPP SystmOne software, the study data will be limited to the questionnaire results, and analysis will take place outside the OpenSAFELY environment. Figure 1 provides an overview of the study.

Follow-up

Participants will be prompted by a push notification (a pop up on their phone screen) to respond to each questionnaire at the time of recruitment, and then at 30, 60 and 90 days after recruitment. The demographic questionnaire will be asked only at baseline (including for example, age, sex, relationship status, socioeconomic status, etc). Participants will be invited to complete all questionnaires, but partially complete data will also be available for analysis.

System reports will flag incomplete questionnaires and one additional push notification reminder will be sent to participants who have not completed any questionnaires within 5 days of the initial notification. Active follow-up will end at 90 days when participants are

For peer review only - http://bmjopen.bmj.com/site/about/guidelines.xhtml

asked to complete their final set of questionnaires. Ongoing, passive follow-up will be continued within the routinely collected electronic health record once the data linkage is in place. At the point of recruitment, participants will be offered the opportunity to consent to linkage and use of their data by researchers.

Participants are free to withdraw from the study at any time and there will be no implications for their care. Withdrawal of consent will occur through Airmid, by moving the opt-in toggle to opt-out.

Study size

The primary research question is to estimate the impact of long COVID on HRQoL as measured using EQ-5D scores at multiple timepoints. Average quality of life in a study of people with severe hospitalised COVID estimated *pre-COVID* HRQoL scores (EQ-5D) to be 0.9655 (standard deviation 0.0958)) and then 0.7054 (standard deviation 0.2514) six months after COVID.[18] We anticipate that people with long COVID participating in the present study will be a mixture of hospitalised and non-hospitalised patients, so are likely to be affected to a lesser extent than a cohort of only hospitalised patients. A second study in a general population cohort estimated EQ-5D scores to be 0.979 (SD 0.0530) among people with no chronic diseases, and 0.9360 (SD 0.112) among those with one chronic disease.[19]

We have therefore performed a power calculation based on the conservative estimates of 0.979 in participants without long COVID, and 0.9360 in those with long COVID (comparable to people with one chronic disease). At the time of calculation, population prevalence of long COVID was estimated to be 2.7%. We assumed that we will over-recruit people with long COVID, due to our focused recruitment strategy. Assuming that 5% of our participants have long COVID, this gives a sample size of 1172 participants to achieve 80% power (alpha 0.05) (corresponding to 1116 in the group without long COVID and 56 among those with long COVID). We intend to keep recruiting beyond this target, as there is no additional cost to the study and minimal risk to the additional respondents, and it will increase our power to address future research questions.

For peer review only - http://bmjopen.bmj.com/site/about/guidelines.xhtml

To explore sampling bias, we will compare characteristics of people registered at GPs using TPP SysmOne software who take part in the study to those registered at other practices, using data from their electronic health record, and additionally compare our results to those of the Office for National Statistics[20] and the Institute for Fiscal Studies[21]

Analysis plan

The main outcome will be QALYs lost after an episode of COVID, calculated from HRQoL as measured by the EuroQOL EQ-5D questionnaire at multiple timepoints. OpenPROMPT will also explore outcomes based on the results from the symptom and productivity questionnaire results.

The main exposure will be long COVID. This will be self-reported by participants in the questionnaires, and recorded in routinely collected data in OpenSAFELY (primary and secondary care data and COVID testing data). The definition of long COVID used in this study will be based on a combination of self-report, diagnoses and referrals for long COVID; the definition will be explored with sensitivity analyses

OpenPROMPT will analyse individual-level determinants of HRQoL deficits in people with long COVID, including key variables such as ethnicity, age, socioeconomic status, and pre-existing health conditions.

This will generate a strong health economic evidence base for assessing the impact of long COVID and evaluating future interventions (including essential inputs to cost-effectiveness analyses of interventions like vaccination boosters, therapeutics and non-pharmaceutical interventions), and demonstrate the effect of long COVID on the health system so far.

Patient and public involvement

We have two streams of patients and public involvement. First, OpenPROMPT recruited a three person steering group, including members with long COVID, those experienced in patient and public involvement or with an interest in long COVID. The steering group aims

to meet with the research team online every 6 months. This group has given input into the design of the study and the planned analysis and will provide further input into the interpretation of research outputs, and to identify and prioritise future goals.

The second strand is broader engagement with research using electronic health records that is being undertaken as part of OpenSAFELY. This includes formation of an Oversight Board (which reviewed OpenPROMPT), publishing blogs explaining OpenSAFELY, presenting results of studies at public and professional events, soliciting feedback on studies via Twitter and email, conducting focus groups. As part of this project, the OpenPROMPT team reviewed existing qualitative studies on living with long COVID and conducted focus groups to explore the goals for the research and the feedback that patients want.

At study close, a summary will be published on the OpenPROMPT website[22] and the study team will give an online interactive webinar describing the results and findings.

Ethics and dissemination

Safety reporting

As this is an observational study, an adverse event in this protocol is defined as any untoward medical occurrence in a patient that has occurred from research interventions only. In this case the research interventions are questionnaires, and the risk to participants is minimal.

Ethical review

The Study Coordination Centre has obtained approval from the LSHTM Research Ethics Committee (ref 28030), as well as a favourable opinion from the South Central – Berkshire B Research Ethics Committee (ref 22/SC/0198). Substantial amendments to the study protocol will obtain a favourable opinion by the local REC before they are implemented.

Dissemination plan

All publications and presentations relating to the study will be authorised by the Study Management Group and also follow the OpenSAFELY publication policy.[23] The first

For peer review only - http://bmjopen.bmj.com/site/about/guidelines.xhtml

publication of the study's results will be in the name of the Study Management Group, if this does not conflict with the journal's policy. If there are named authors, these will include at least the trial's Chief Investigator, Statistician and Study Coordinator. Members of the Study Management Group will be listed and contributors will be cited by name if published in a journal where this does not conflict with the journal's policy. Authorship of parallel studies initiated outside of the Study Management Group will be according to the individuals involved in the project but must acknowledge the contribution of the Study Management Group and the Study Coordination Centre.

All publications resulting from the OpenPROMPT study will be open access. Pre-prints will be made available prior to peer review.

Data sharing

An aggregated version of the data will be included in publications arising from the study. The questionnaire data provided by participants will be made available to allow other researchers to benefit from this work in the future, and for a range of different studies and purposes. Opting-in to the study will require an affirmative response to agree to use of data in this way.

After completion of the study, a pseudonymised copy of the data will be held according to NHS England retention policy.[24] Data access will be governed by LSHTM and will require researchers to complete a data access form. This will be explained in all publications arising from the study.

All other study documentation will be stored for a minimum of five years after the follow-up period.

Authors' contributions

RE, AB, BG, LS, MM, FS, JP, CB, JM, MJ, LT and SB conceived the study. EH, RE, KT, and LYL refined the design and the protocol with contributions from RE, AB, BG, LS, MM, FS, JP, CB,

For peer review only - http://bmjopen.bmj.com/site/about/guidelines.xhtml

JM, MJ, LT, SB, AD, DS, EG, BB-C, VM and AM. All authors reviewed have reviewed and commented on the protocol and have approved this final version for publication.

Competing interests statement

All authors have completed the ICMJE uniform disclosure form at www.icmje.org/coi_disclosure.pdf and declare the following: BG has received research funding from the Laura and John Arnold Foundation, the NHS National Institute for Health Research (NIHR), the NIHR School of Primary Care Research, NHS England, the NIHR Oxford Biomedical Research Centre, the Mohn-Westlake Foundation, NIHR Applied Research Collaboration Oxford and Thames Valley, the Wellcome Trust, the Good Thinking Foundation, Health Data Research UK, the Health Foundation, the World Health Organisation, UKRI MRC, Asthma UK, the British Lung Foundation, and the Longitudinal Health and Wellbeing strand of the National Core Studies programme; he is a Non-Executive Director at NHS Digital; he also receives personal income from speaking and writing for lay audiences on the misuse of science. DS has a zero hours contract with NHS Digital, NHS Health Education West Midlands and the Usher Institute at Edinburgh University, and is part of the patient panel for the British Society of Immunology and the Wales COVID Evidence Centre. MJ has received funding from NIHR, MRC, Wellcome Trust, BMGF, WHO, Gavi and US CDC.

Funding statement

This work was supported by the NIHR Long COVID grant number COV-LT2-0073. This work was jointly funded by UK Research and Innovation (UKRI) (COV0076;MR/V015737/1), the Longitudinal Health and Wellbeing strand of the National Core Studies programme (MC_PC_20030: MC_PC_20059: COV-LT-0009), National Institute for Health Research (NIHR), and Asthma UK-British Lung Foundation (BLF). This research will also use data assets made available as part of the Data and Connectivity National Core Study, led by Health Data Research UK in partnership with the Office for National Statistics and funded by UK Research and Innovation (grant ref MC_PC_20058). In addition, the OpenSAFELY Platform is supported by grants from the Wellcome Trust (222097/Z/20/Z); MRC (MR/V015757/1, MC_PC-20059, MR/W016729/1); NIHR (NIHR135559), and Health Data Research UK

For peer review only - http://bmjopen.bmj.com/site/about/guidelines.xhtml

(HDRUK2021.000, 2021.0157). EH was funded by an NIHR post-doctoral fellowship (PDF-2016-09-029). VM was funded by a NIHR fellowship (NIHR301535).

The views expressed are those of the authors and not necessarily those of the NIHR, NHS England, Public Health England, or the Department of Health and Social Care. The funders had no role in the study design, in the writing of the report and in the decision to submit the article for publication.

References

1. Franco, J. V. A. *et al.* Long-Term Health Symptoms and Sequelae Following SARS-CoV-2 Infection: An Evidence Map. *Int J Environ Res Public Health* **19**, 9915 (2022).
2. Overview | COVID-19 rapid guideline: managing the long-term effects of COVID-19 | Guidance | NICE. https://www.nice.org.uk/guidance/ng188.
3. Al-Aly, Z., Xie, Y. & Bowe, B. High-dimensional characterization of post-acute sequelae of COVID-19. *Nature* **594**, 259–264 (2021).
4. Jacobson, K. B. *et al.* Patients With Uncomplicated Coronavirus Disease 2019 (COVID-19) Have Long-Term Persistent Symptoms and Functional Impairment Similar to Patients with Severe COVID-19: A Cautionary Tale During a Global Pandemic. *Clin Infect Dis* **73**, e826–e829 (2021).
5. Whittaker, H. *et al.* Post-Acute COVID-19 Sequelae in Cases Managed in the Community or Hospital in the UK: A Population Based Stud. Preprint at https://doi.org/10.2139/ssrn.3820510 (2021).
6. Prevalence of ongoing symptoms following coronavirus (COVID-19) infection in the UK - Office for National Statistics.

For peer review only

https://www.ons.gov.uk/peoplepopulationandcommunity/healthandsocialcare/conditionsanddiseases/bulletins/prevalenceofongoingsymptomsfollowingcoronaviruscovid19infectionintheuk/3november2022.

7. de Oliveira Almeida, K. *et al.* A systematic review on physical function, activities of daily living and health-related quality of life in COVID-19 survivors. *Chronic Illn* 17423953221089308 (2022) doi:10.1177/17423953221089309.

8. Malik, P. *et al.* Post-acute COVID-19 syndrome (PCS) and health-related quality of life (HRQoL)-A systematic review and meta-analysis. *J Med Virol* **94**, 253–262 (2022).

9. Nandasena, H. M. R. K. G., Pathirathna, M. L., Atapattu, A. M. M. P. & Prasanga, P. T. S. Quality of life of COVID 19 patients after discharge: Systematic review. *PLOS ONE* **17**, e0263941 (2022).

10. Antonelli, M., Pujol, J. C., Spector, T. D., Ourselin, S. & Steves, C. J. Risk of long COVID associated with delta versus omicron variants of SARS-CoV-2. *The Lancet* **399**, 2263–2264 (2022).

11. Bowe, B., Xie, Y. & Al-Aly, Z. Acute and postacute sequelae associated with SARS-CoV-2 reinfection. *Nat Med* **28**, 2398–2405 (2022).

12. Airmid. *TPP* https://tpp-uk.com/products/airmid/ (2020).

13. EQ-5D. https://euroqol.org/.

14. Reilly, M. Work Productivity and Activity Impairment Questionnaire: 2.

15. MRC Dyspnoea Scale | Primary Care Respiratory Society. https://www.pcrs-uk.org/mrc-dyspnoea-scale.

16. FACIT-F. *FACIT Group* https://www.facit.org/measures/FACIT-F.

17. OpenSAFELY: Representativeness of ... | Wellcome Open Research. https://wellcomeopenresearch.org/articles/7-191/v1.

For peer review only

18. Taboada, M. *et al.* Quality of life, functional status, and persistent symptoms after intensive care of COVID-19 patients. *Br J Anaesth* **126**, e110–e113 (2021).

19. Ping, W. *et al.* Evaluation of health-related quality of life using EQ-5D in China during the COVID-19 pandemic. *PLOS ONE* **15**, e0234850 (2020).

20. Prevalence of ongoing symptoms following coronavirus (COVID-19) infection in the UK - Office for National Statistics. https://www.ons.gov.uk/peoplepopulationandcommunity/healthandsocialcare/conditionsanddiseases/bulletins/prevalenceofongoingsymptomsfollowingcoronaviruscovid19infectionintheuk/7april2022.

21. Long COVID and the labour market. *Institute for Fiscal Studies* https://ifs.org.uk/publications/long-covid-and-labour-market.

22. OpenSAFELY long COVID study website. https://www.opensafely.org/studies/long-covid/.

23. OpenSAFELY: Policies for Researchers. https://www.opensafely.org/policies-for-researchers/.

24. NHS England » OpenSAFELY – the Coronavirus (COVID-19) Research Platform. https://www.england.nhs.uk/contact-us/privacy-notice/how-we-use-your-information/covid-19-response/coronavirus-covid-19-research-platform/.

Word Count
2749

Figure legends

Figure 1. Overview of study

For peer review only

For peer review only - http://bmjopen.bmj.com/site/about/guidelines.xhtml

For peer review only

For peer review only - http://bmjopen.bmj.com/site/about/guidelines.xhtml

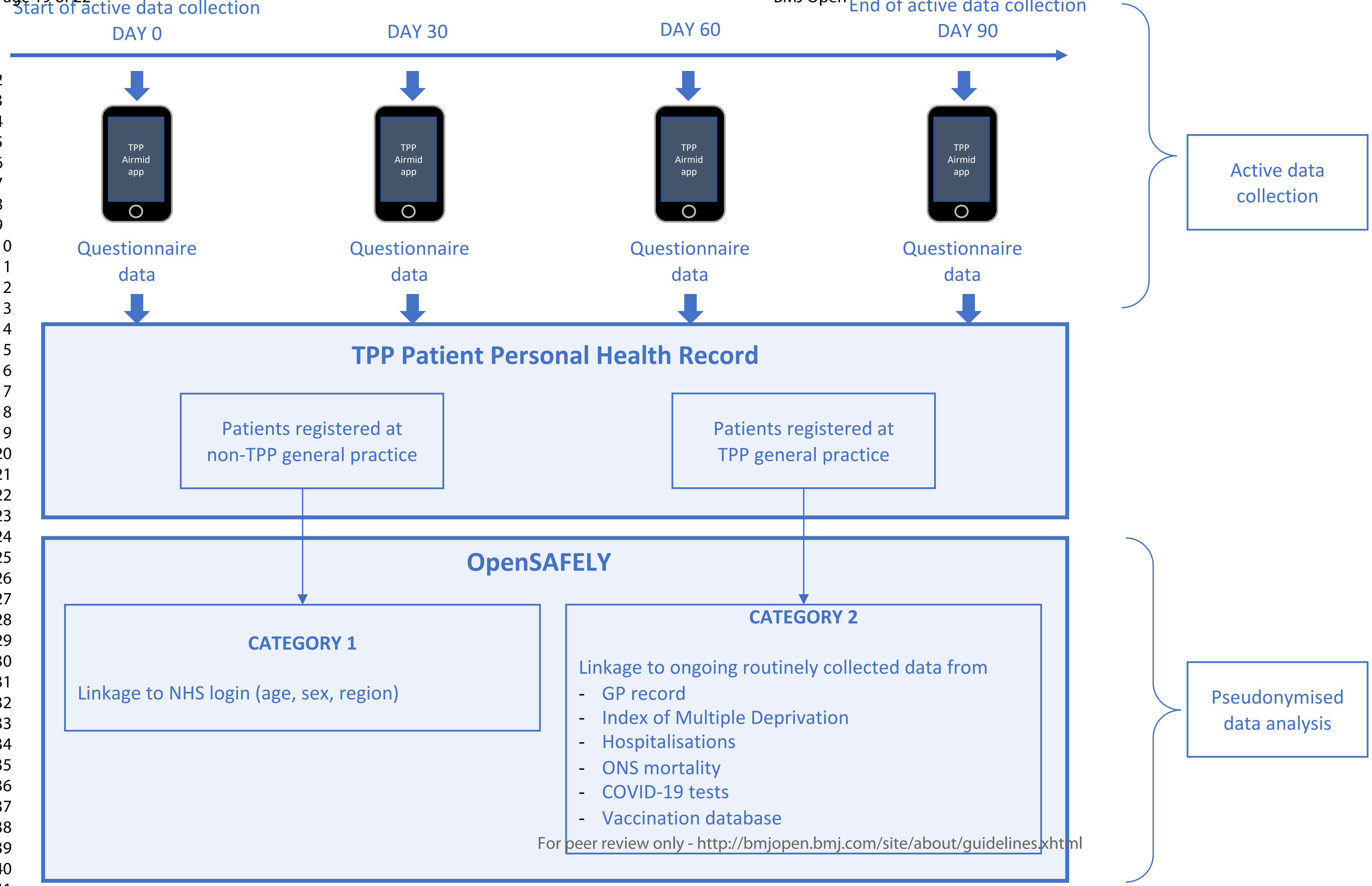
Start of active data collection
DAY 0
DAY 30
DAY 60
End of active data collection
DAY 90
TPP Airmid app
TPP Airmid app
TPP Airmid app
TPP Airmid app
Questionnaire data
Questionnaire data
Questionnaire data
Questionnaire data
Active data collection
TPP Patient Personal Health Record
Patients registered at non-TPP general practice
Patients registered at TPP general practice
OpenSAFELY
CATEGORY 1
Linkage to NHS login (age, sex, region)
CATEGORY 2
Linkage to ongoing routinely collected data from
- GP record
- Index of Multiple Deprivation
- Hospitalisations
- ONS mortality
- COVID-19 tests
- Vaccination database
Pseudonymised data analysis

Reporting checklist for cohort study.

Based on the STROBE cohort guidelines.

Instructions to authors

Complete this checklist by entering the page numbers from your manuscript where readers will find each of the items listed below.

Your article may not currently address all the items on the checklist. Please modify your text to include the missing information. If you are certain that an item does not apply, please write "n/a" and provide a short explanation.

Upload your completed checklist as an extra file when you submit to a journal.

In your methods section, say that you used the STROBE cohortreporting guidelines, and cite them as:

von Elm E, Altman DG, Egger M, Pocock SJ, Gotzsche PC, Vandenbroucke JP. The Strengthening the Reporting of Observational Studies in Epidemiology (STROBE) Statement: guidelines for reporting observational studies.

For peer review only

		Reporting Item	Page Number
**Title and abstract**			
Title	#1a	Indicate the study's design with a commonly used term in the title or the abstract	1
Abstract	#1b	Provide in the abstract an informative and balanced summary of what was done and what was found	2
**Introduction**			
Background / rationale	#2	Explain the scientific background and rationale for the investigation being reported	4
Objectives	#3	State specific objectives, including any prespecified hypotheses	5
**Methods**			
Study design	#4	Present key elements of study design early in the paper	5-6

Setting	#5	Describe the setting, locations, and relevant dates, including periods of recruitment, exposure, follow-up, and data collection	5-6
Eligibility criteria	#6a	Give the eligibility criteria, and the sources and methods of selection of participants. Describe methods of follow-up.	6
Eligibility criteria	#6b	For matched studies, give matching criteria and number of exposed and unexposed	n/a
Variables	#7	Clearly define all outcomes, exposures, predictors, potential confounders, and effect modifiers. Give diagnostic criteria, if applicable	7 - questionnaires
Data sources / measurement	#8	For each variable of interest give sources of data and details of methods of assessment (measurement). Describe comparability of assessment methods if there is more than one group. Give information separately for for exposed and unexposed groups if applicable.	7
Bias	#9	Describe any efforts to address potential sources of bias	10
Study size	#10	Explain how the study size was arrived at	9-10
Quantitative variables	#11	Explain how quantitative variables were handled in the analyses. If applicable, describe which groupings were chosen, and why	n/a
Statistical methods	#12a	Describe all statistical methods, including those used to control for confounding	n/a - protocol
Statistical methods	#12b	Describe any methods used to examine subgroups and interactions	n/a protocol
Statistical methods	#12c	Explain how missing data were addressed	n/a - protocol
Statistical methods	#12d	If applicable, explain how loss to follow-up was addressed	n/a - protocol
Statistical methods	#12e	Describe any sensitivity analyses	

For peer review only

		which confounders were adjusted for and why they were included	
Main results	#16b	Report category boundaries when continuous variables were categorized	n/a protocol
Main results	#16c	If relevant, consider translating estimates of relative risk into absolute risk for a meaningful time period	n/a protocol
Other analyses	#17	Report other analyses done—eg analyses of subgroups and interactions, and sensitivity analyses	n/a protocol
**Discussion**			
Key results	#18	Summarise key results with reference to study objectives	n/a protocol
Limitations	#19	Discuss limitations of the study, taking into account sources of potential bias or imprecision. Discuss both direction and magnitude of any potential bias.	n/a protocol
Interpretation	#20	Give a cautious overall interpretation considering objectives, limitations, multiplicity of analyses, results from similar studies, and other relevant evidence.	n/a protocol
Generalisability	#21	Discuss the generalisability (external validity) of the study results	n/a protocol
**Other Information**			
Funding	#22	Give the source of funding and the role of the funders for the present study and, if applicable, for the original study on which the present article is based	15

None The STROBE checklist is distributed under the terms of the Creative Commons Attribution License CC-BY. This checklist can be completed online using https://www.goodreports.org/, a tool made by the EQUATOR Network in collaboration with Penelope.ai

For peer review only
