# Supplementary material for: Protocol for an OpenSAFELY cohort study collecting patient-reported outcome measures using the TPP Airmid smartphone application and linked big data to quantify the health and economic costs of long COVID (OpenPROMPT)
Source: BMJ Open. 2023 Feb 17;13(2):e071261. doi: 10.1136/bmjopen-2022-071261 (PMC9943695; doi:10.1136/bmjopen-2022-071261)
Supplement: Reviewer comments [file bmjopen-2022-071261.reviewer_comments.pdf]

PEER REVIEW HISTORY

BMJ Open publishes all reviews undertaken for accepted manuscripts. Reviewers are asked to complete a checklist review form (**http://bmjopen.bmj.com/site/about/resources/checklist.pdf**) and are provided with free text boxes to elaborate on their assessment. These free text comments are reproduced below.

ARTICLE DETAILS

**TITLE (PROVISIONAL)**	Protocol for an OpenSAFELY cohort study collecting Patient Reported Outcome Measures using the TPP Airmid smartphone application and linked big data to quantify the health and economic costs of long COVID (OpenPROMPT)
**AUTHORS**	Herrett, Emily; Tomlin, Keith; Lin, Liang-Yu; Tomlinson, Laurie; Jit, Mark; Briggs, Andrew; Marks, Michael; Sandmann, Frank; Parry, John; Bates, Christopher; Morley, Jessica; Bacon, Seb; Butler-Cole, Benjamin; Mahalingasivam, Viyaasan; Dennison, Alan; Smith, Deb; Gabriel, Ethan; Mehrkar, Amir; Goldacre, Ben; Smeeth, Liam; Eggo, Rosalind M

This article was not externally reviewed at BMJ Open. Protocols that have been independently assessed prior to submission to BMJ Open are usually fast-tracked to publication on the grounds that further substantial changes will not be possible. This independent assessment will usually be external, independent review for both a substantial grant award from a non-commercial or government funder and ethics approval.
